# Real-Time fMRI Neurofeedback in Patients With Tobacco Use Disorder During Smoking Cessation: Functional Differences and Implications of the First Training Session in Regard to Future Abstinence or Relapse

**DOI:** 10.3389/fnhum.2019.00065

**Published:** 2019-03-04

**Authors:** Susanne Karch, Marco Paolini, Sarah Gschwendtner, Hannah Jeanty, Arne Reckenfelderbäumer, Omar Yaseen, Maximilian Maywald, Christina Fuchs, Boris-Stephan Rauchmann, Agnieszka Chrobok, Andrea Rabenstein, Birgit Ertl-Wagner, Oliver Pogarell, Daniel Keeser, Tobias Rüther

**Affiliations:** ^1^Department of Psychiatry and Psychotherapy, University Hospital, Ludwig Maximilian University of Munich, Munich, Germany; ^2^Department of Radiology, University Hospital, Ludwig Maximilian University of Munich, Munich, Germany

**Keywords:** real-time fMRI, neurofeedback, craving, tobacco use disorder, therapy success

## Abstract

One of the most prominent symptoms in addiction disorders is the strong desire to consume a particular substance or to show a certain behavior (craving). The strong association between craving and the probability of relapse emphasizes the importance of craving in the therapeutic process. Former studies have demonstrated that neuromodulation using real-time fMRI (rtfMRI) neurofeedback (NF) can be used as a treatment modality in patients with tobacco use disorder. The aim of the present project was to determine whether it is possible to predict the outcome of NF training plus group psychotherapy at the beginning of the treatment. For that purpose, neuronal responses during the first rtfMRI NF session of patients who remained abstinent for at least 3 months were compared to those of patients with relapse. All patients were included in a certified smoke-free course and took part in three NF sessions. During the rtfMRI NF sessions tobacco-associated and neutral pictures were presented. Subjects were instructed to reduce their neuronal responses during the presentation of smoking cues in an individualized region of interest for craving [anterior cingulate cortex (ACC), insula or dorsolateral prefrontal cortex]. Patients were stratified to different groups [abstinence (*N* = 10) vs. relapse (*N* = 12)] according to their individual smoking status 3 months after the rtfMRI NF training. A direct comparison of BOLD responses during the first NF-session of patients who had remained abstinent over 3 months after the NF training and patients who had relapsed after 3 months showed that patients of the relapse group demonstrated enhanced BOLD responses, especially in the ACC, the supplementary motor area as well as dorsolateral prefrontal areas, compared to abstinent patients. These results suggest that there is a probability of estimating a successful withdrawal in patients with tobacco use disorder by analyzing the first rtfMRI NF session: a pronounced reduction of frontal responses during NF training in patients might be the functional correlate of better therapeutic success. The results of the first NF sessions could be useful as predictor whether a patient will be able to achieve success after the behavioral group therapy and NF training in quitting smoking or not.

## Introduction

Smoking tobacco can lead to diverse symptoms and illnesses including cancer, respiratory and cardiovascular diseases and is one of the most significant causes of death in Europe (Ezzati and Lopez, 2003; Sasco et al., 2004; Thun et al., 2013). Worldwide more than 5 million people per year die as a result of tobacco use^1^ (World Health Organization [WHO], 2018). In addition, tobacco users who die prematurely deprive their families of income, raise the cost of health care and slow down the economic development^1^ (World Health Organization [WHO], 2018).

Important aspects of tobacco use disorder are a reduced control of tobacco intake, the inability to stop or reduce substance use, tolerance development, withdrawal symptoms and a strong desire to consume the particular substance (craving behavior). Even though more than 70% of smokers want to quit, only 5% are successful in doing so (Hatsukami et al., 2008). In addition, the relapse rate in patients with tobacco addiction is relatively high. According to different meta-analyses there is not enough evidence that behavioral therapies alone can prevent long-term relapse (Agboola et al., 2010; Hajek et al., 2013). Even with combined medication and cognitive behavioral therapies, the most common outcome 1 year after an attempt to quit is a relapse (Piasecki, 2006). A review about the effectiveness of different medication therapies combined with behavioral support in the United Kingdom showed that abstinence rates were comparable after 3 months with mean values of pooled point prevalence between 35 and 55% (Coleman et al., 2010). The common German cognitive behavioral therapy program called “Rauchfrei Programm” (translation: smoke-free program) showed an abstinence rate of 40% after 6 months and 31% after 1 year (Gradl et al., 2009; Wenig et al., 2013). Therefore, the need for new and improved treatments helping smokers to stop smoking seems obvious. Craving can be elicited, e.g., by the Presentation of Nicotine-Relevant Information (Saladin et al., 2012). The regional areas of brain activation associated with craving in nicotine-dependent smokers are scientifically well studied. Functional neuroimaging studies have examined increased craving-related responses, e.g., in the anterior cingulate cortex (ACC) (McClernon et al., 2005; Wilson et al., 2005; Brody et al., 2007; Goudriaan et al., 2010; Hartwell et al., 2011), the medial prefrontal cortex (mPFC) (Hartwell et al., 2011) and the precuneus/cuneus (Smolka et al., 2006; Hartwell et al., 2011) during the presentation of substance-related information, while these areas are linked to attentional processes (Augustus Diggs et al., 2013) and motivation (Augustus Diggs et al., 2013). Also, the insula has shown to play a major role in addictive behavior (Naqvi and Bechara, 2010). The role of the insula is not yet clear: it may be related to conscious interoception, emotional experience and decision-making. Naqvi and colleagues presented evidence that the insula represents the interoceptive effects of drug taking, making this information available to conscious awareness, memory and executive functions (Naqvi and Bechara, 2010; Naqvi et al., 2014). In addition, the orbitofrontal cortex (OFC) (Hartwell et al., 2011) which is thought to be related to cognitive reappraisal (Goldstein and Volkow, 2002) and regions involved in decision making and goal-directed behavior such as the dorsolateral prefrontal cortex (DLPFC) (Goldstein and Volkow, 2002) seem to be important.

Results of the meta-analysis focusing on neurobiological aspects of smoking cue reactivity in smokers indicate that smoking cues reliably evoke larger neuronal responses than neutral cues in the extended visual system, the precuneus, the posterior cingulate gyrus, the ACC, the dorsal prefrontal cortex (dPFC) and the mPFC, the insula, and the dorsal striatum (Engelmann et al., 2012). The areas that were found to be responsive to smoking cues agree in most parts with theories of the neurobiology of cue reactivity (Engelmann et al., 2012). Surprisingly, there was a reliable cue reactivity effect in the precuneus which is not typically considered a brain region important to addiction (Engelmann et al., 2012). Furthermore, the meta-analysis did not show any significant effect in the nucleus accumbens (Engelmann et al., 2012). Altogether, the authors of the meta-analysis emphasize that the extended visual system should receive more attention in future studies of smoking cue reactivity (Engelmann et al., 2012).

A good overview of cue-related activities and their functions has been presented by Miller (2013). They report that neuronal responses which are related to cues have been shown in brain regions that are associated with attention, reward and goal-directed behavior (Miller, 2013). Responses in the secondary and tertiary visual cortex, the precuneus as well as the gyrus fusiformis have been observed (Miller, 2013). Activations in these regions show an increased allocation of attention on the visual smoking cues (Miller, 2013). Activations of limbic and paralimbic structures including the hippocampus, the thalamus, the amygdala, the insula and the cingulate cortex reflect the contribution of emotional processes (Miller, 2013). Moreover, increased cue-related responses have been shown in the posterior cingulate cortex and particularly in the ACC (Miller, 2013). Activations in the ACC have been interpreted as reflecting, e.g., conflict monitoring and reward learning, as well as the emotional relevance of stimuli (Miller, 2013). Motivational processes of smoking-related cues have been linked to BOLD responses in the ventral tegmentum and the ventral striatum (Miller, 2013). In addition, BOLD responses in the PFC including the orbitofrontal cortex (OFC), the inferior frontal gyrus (IFG), the medial frontal gyrus (MFG) and the superior frontal gyrus (SFG) have been related to emotion and reward-related processes (OFC and IFG) and the mobilization of cognitive control and executive processes (MFG and SFG) (Miller, 2013).

Some researchers have investigated a possible difference between ‘craving’ and ‘resisting craving’ regarding the underlying neuronal brain regions. For example the ACC could be especially detected for ‘craving’ whereas ‘resisting craving’ was more assigned to the dorsomedial PFC (Hanlon et al., 2013). The cigarette cue resist condition elicited enhanced brain responses in the left dorsal ACC, the posterior cingulate cortex (PCC), and the precuneus compared with the cigarette cue crave condition (Brody et al., 2007). In addition, a study which focused on craving and resisting craving found a considerable overlap between the areas activated during craving and attempts to resist craving, supporting the idea that these two aspects do not have to be investigated separately because craving is almost always associated with some degree of resisting the urge to smoke, and vice versa (Hartwell et al., 2011).

Functional magnetic resonance imaging also enables the detection of functional connectivity which has been shown to be altered in several neurodegenerative and neuropsychiatric diseases, including addiction disorders. Concerning nicotine addiction, smokers show a loss of functional connectivity in brain areas of the executive control network and an increase of connectivity in brain areas of the default mode network modulated by the insula, or the salience network which contains the insula (Fedota and Stein, 2015; Vergara et al., 2017). Furthermore, the thalamostriatal connectivity seems to be increased in smokers. Recent prediction studies showed that the risk of relapse increases when addicted patients have decreased functional connectivity in corticolimbic and corticostriatal networks (Moeller and Paulus, 2018). In tobacco dependent people insula-related functional connectivity seems to be positively correlated with the success in smoking cessation. In this context connectivity between the insula and primary sensorimotor cortical areas or control-related brain regions, such as the dACC and the DLPFC, seems to play an important role in the potential to stop smoking and to stay abstinent (Janes et al., 2010; Addicott et al., 2015; Zelle et al., 2017).

Several studies focused on the relationship between craving and relapse rates. A systematic review showed mixed results with respect to the relationship between craving and relapse rate (Wray et al., 2013). By contrast, a recent study demonstrated that greater neural activation during pre-treatment exposure to smoking cues in the right ventral striatum, the left amygdala, and the anterior cingulate was associated with longer periods of abstinence following cessation (Owens et al., 2017). The authors concluded that these results suggest that pre-treatment reactivity to smoking cues in areas associated with cue reactivity may be associated with successfully maintaining abstinence during treatment (Owens et al., 2017). Another study demonstrated that subjects that were not successful in their attempt to quit smoking revealed heightened fMRI reactivity to smoking-related images in brain regions implicated in emotion, interoceptive awareness, and motor planning and execution (Janes et al., 2010). Additionally, these subjects had decreased functional connectivity between a network comprising the insula and brain regions involved in cognitive control (Janes et al., 2010). Overall there is some evidence for a relevant association between craving and/or craving-related neurobiological responses and the risk of relapse. This emphasizes its importance for future studies and within the therapeutic process. Hence, reducing craving and the physiological response to smoking cues could well have positive effects on smoking cessation outcomes.

Neurofeedback (NF) delivered via real-time functional magnetic resonance imaging (rtfMRI) enables the immediate visualization of brain activations or functional connectivity between brain areas, and offers the possibility to modulate voluntarily neuronal activity in circumscribed brain areas. It can also be seen as a training method whereby a person is confronted with a mental or emotional task while simultaneously receiving information about changes in neural activity in brain areas. This information can be used for self-regulation, control and modulation of the neural activity in a target region which is important for the ongoing task. The modulation of neuronal responses is expected to lead to changing behavior (Stoeckel et al., 2014). It is assumed that predominantly implicit learning processes, including operant conditioning, modulate distinct behavioral patterns (Caria et al., 2007; deCharms, 2007).

While EEG-based NF is restricted to the modulation of neuronal activity in cortical areas as well as relatively broad brain regions, fMRI-based NF can be used to modulate the activity in subcortical areas and small cortical brain regions, as well as functional connectivity between areas (Koush et al., 2017). Several studies refer to brain regions which are related to emotional and/or cognitive processes (Posse et al., 2003; Caria et al., 2007; Johnston et al., 2010; Dyck et al., 2011; Hamilton et al., 2011; Lee et al., 2011; Zotev et al., 2011). Other studies demonstrate the modulation leading to specific behavioral effects (Rota et al., 2009; Caria et al., 2010).

There is already some evidence about positive effects of EEG, respectively, fMRI NF training on patients with attention-deficit/hyperactivity disorder (ADHD) (Arnold et al., 2013; Zilverstand et al., 2017), as well as depression (Choi et al., 2011; Linden et al., 2012). RtfMRI studies in schizophrenic patients have shown that patients were able to influence their insular activity (Ruiz et al., 2013a,b). Functional variations were accompanied by an improvement to recognize negative facial expressions (Ruiz et al., 2013a). In depressive patients training of brain regions which are associated with emotion regulation was related to an improvement of depressive symptomatology (Linden et al., 2012).

There are only a few studies focusing on NF processes in persons with addiction-related symptoms (Hartwell et al., 2013). However, there are a lot of indices that neuromodulation can be a unique opportunity to directly apply neuroscientific knowledge to the treatment of addiction (Luigjes et al., 2013). Kirsch et al. (2016) examined the modulation of reward-related striatal brain responses in non-addicted heavy social drinkers: subjects were instructed to downregulate the responses in their ventral striatum. RtfMRI led to a significant downregulation of striatal activations in the real group, whereby the sham conditions did not reveal comparable effects (Kirsch et al., 2016). A study of our own working group aimed at modulating craving-associated neuronal responses in patients with alcohol addiction using individualized feedback (Karch et al., 2015). The results showed a significant reduction of neuronal responses in patients at the end of the training compared to the beginning, especially in the ACC, the insula, the inferior temporal gyrus and the medial frontal gyrus. In addition, patients reported slightly reduced craving after the NF training, compared to before. The results suggest that it is feasible for patients with alcohol dependency to reduce their neuronal activity using rtfMRI NF (Karch et al., 2015).

Regarding nicotine dependence, Li et al. (2013) demonstrated that smokers with rtfMRI NF were able to reduce voluntarily neuronal responses in the ACC during the presentation of smoking-relevant information. These modulations were associated with a temporarily decreased craving for nicotine (Li et al., 2013). Hartwell et al. (2016) showed that individualized real-time fMRI NF can be an appropriate method to attenuate craving in nicotine-dependent smokers (Hartwell et al., 2016). The efficacy of multiple NF training sessions as well as the need to consider the nicotine-dependence severity was further supported by the fact that individuals with lower nicotine-dependence severity were more successful in reducing the activation in the ACC over time (Canterberry et al., 2013). Recent studies mention that the additional inclusion of functional connectivity information in fMRI-based NF could improve its efficacy in the reduction of cigarette craving (Kim et al., 2015). Overall, rtfMRI NF has been increasingly discussed as a potential therapeutic method (Augustus Diggs et al., 2013; Bruhl, 2015; Sitaram et al., 2017; Sokunbi, 2017).

Especially because of the comparatively great effort for patients, the high technical requirements and the high costs of rtfMRI NF training, it seems to be relevant to find indicators for a therapeutic indication. Former studies regarding the prediction of smoking relapse suggest that smokers high in anger trait may have greater mood difficulties during abstinence and may be more vulnerable to early relapse than smokers with low anger trait (al’Absi et al., 2007). Another study found differences in cue reactivity of smokers before participating in a cessation clinical trial predicting outcomes with 79% accuracy in combination with results of an Emotional Stroop task (Janes et al., 2010). In a resting state fMRI study, a logistic regression based on functional connectivity predicted relapse of smokers before medication therapy with 80.7% accuracy (Shen et al., 2017). Classifying abstinent smokers according to their individual relapse risk profile may be helpful in order to find the best therapeutic strategy, for example to switch to medication therapy or to modulate the existing strategy, or to even intensify the neurofeedback training. In this context, the use of neuroimaging data for prediction models seems to be promising for addiction disorders in general as fMRI data show altered brain reactivity to drug-related and non-drug-related cues and certain changes in functional connectivity and gray and white matter volumes (Moeller and Paulus, 2018). Concerning fMRI neurofeedback, there is no data as yet about the prediction of abstinent smokers regarding the risk of relapse.

The aim of the present project was to determine whether it is possible to predict functional differences of patients who remained abstinent and patients who relapsed after receiving rtfMRI NF training plus group psychotherapy. We focused especially on the question whether there are any brain activity differences between groups which appear already at the beginning of NF training. For that purpose, patients were stratified in two separate groups according to their individual treatment success 3 months after the NF training sessions. Neuronal responses during the first rtfMRI NF session of patients who then remained abstinent for at least 3 months were compared to those of patients with relapse. To our knowledge, none of the previous studies has combined NF training with behavioral group therapeutic strategies in patients with tobacco use disorder.

## Materials and Methods

### Subjects

The study comprised the investigation of 54 patients with tobacco use disorder (♀ = 22, ♂ = 32). All patients were recruited through an advertisement in a regional daily newspaper. Key inclusion criteria were age between 18 and 65 years, no prior head injury or lifetime diagnosis of a neurological and/or psychiatric disorder, and the ICD-10 diagnosis of nicotine dependence (*F* 17.2). The exclusion criteria were, e.g., claustrophobia, pregnancy, any implanted metal or a cardiac pacemaker. All participants reported having a solid mental and physical constitution at the time of testing. The study received approval from the local research ethics committee of the Medical Faculty of LMU Munich and is in accordance with the Declaration of Helsinki and subsequent revisions. The participation in the rtfMRI sessions was compensated with 50€ per session. The participation in the group therapeutic program was free of charge.

After proving their study qualification by a short standardized questionnaire in a telephone interview, patients participated in a specialized therapeutic program for nicotine-dependence (“Das Rauchfrei Programm,” IFT – Gesundheitsförderung Gesellschaft mbH, München, 2012) at the Department of Psychiatry and Psychotherapy, LMU Munich. RtfMRI NF training was provided three times as an add-on to the group therapy sessions. Standardized questionnaires were used in order to assess sociodemographic data, information about smoking and craving as well as psychopathological information.

18 patients had to be excluded from the study because of missing measurement appointments or technical problems (four patients), permanent makeup (one patient), dropping out of the group therapeutic program (six patients), structural anatomic brain abnormalities (two patients), medication because of clinical diagnosed depression (one patient) and deviant social behavior (one patient). Three more patients were not available in the follow-up telephone interview; for this reason, it was not possible to assign these patients to one of the groups (abstinence vs. relapse).

Taking into account the exclusions from the study, the results of 36 nicotine-dependent smokers (♀ = 11, ♂ = 25) aged between 19 and 65 years (*M* = 43.83, *SD* = 12.37) with a number of 3 to 51 pack-years (*M* = 26.29, *SD* = 14.43) were analyzed.

All nicotine-dependent smokers were randomized in a real NF training group (*N* = 22) and a sham NF group (*N* = 14). During the real condition, neuronal responses in a ROI that is located in an individual, craving-related area within the insula, the ACC or the DLPFC were presented parallel to the tobacco-associated pictures. During the sham condition, the participants received feedback about neuronal responses in brain areas that are not related to craving (e.g., parietal cortex).

In order to determine the significance of the first NF session for the therapeutic outcome after the complete NF training, we focus in this manuscript on the results of the real group. The results of the sham group will be presented elsewhere in detail (Karch et al., unpublished).

For that purpose in the present study the results of smokers of the real group who remained abstinent 3 months after the rtfMRI NF training and group therapy (abstinent group; *N* = 10) with those of smokers of the real group who relapsed within the first 3 months after the interventions (relapse group; *N* = 12) were compared.

### Psychometric Questionnaires

Different psychometric tests were used as a screening for neurologic and/or psychiatric diseases. The symptomatology of the participants was determined using the Fagerström Test for Nicotine Dependence (FTND) (Heatherton et al., 1991) and Questionnaire on Smoking Urges – German (QSU-G) (Tiffany and Drobes, 1991). Verbal intelligence was assessed using the verbal intelligence test (WST) (Schmidt and Metzler, 1992). In addition, we used several questionnaires in order to determine affective symptoms including the Barratt Impulsiveness Scale (BIS-11) (Patton et al., 1995), the Aggression Questionnaire (AQ) (Buss and Perry, 1992), Beck Depressions Inventar (BDI) (Beck and Steer, 1987), State-Trait-Anger Expression Inventory (STAXI) (Schwenkmezger et al., 1992), State-Trait-Anxiety Inventory (STAI) (Laux et al., 1981), NEO-Five Factor Inventory (NEO-FFI) (Costa and McCrae, 1992).

### Paradigm

**FIGURE 1:**
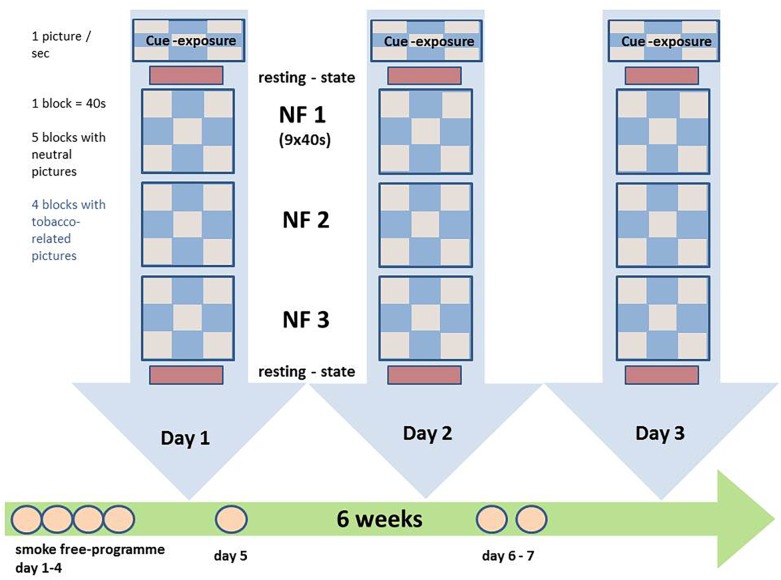
Experimental procedures: the patients participated in three rtfMRI NF sessions within 5 weeks; during the NF training presentation of neutral and tobacco-related pictures in blocks of 40 s with 10 pictures of the respective category; participants were instructed to reduce brain activity during the presentation of tobacco-associated information; during the presentation of neutral information, participants were instructed to simply gaze at the pictures. Before and after each NF training session, resting-state activity was acquired. NF, neurofeedback.

FMRI measurements took place at the Department of Radiology, Ludwig Maximilian University of Munich. The three NF-training sessions were conducted after day 4, day 5, and day 6 or 7 of the smoking-free program (see Figure 1). Before and after each fMRI session participants’ degree of craving was examined with the German version of the “Questionnaire on Smoking Urges” (QSU-G). CO-levels were measured using the UBLOW CO breath tester (Neomed Medizintechnik GmbH).

The visual stimulation utilized consisted of 20 neutral and 20 tobacco-related pictures. The neutral pictures originated from the International Affective Picture System (IAPS^2^) or were taken in the course of the study. Tobacco-relevant pictures contained specific triggers for tobacco consumption, e.g., persons smoking, cigarettes or cigarette packets. The tobacco-related pictures were taken from databases or in the course of the study.

Three paradigms were used during the fMRI measurements: (1) cue exposure, (2) resting state and (3) rtfMRI NF paradigm.

(1) *Cue exposure*: The cue exposure paradigm was used as functional localiser. Neutral and nicotine-related pictures were presented block-wise to the participants using the software program PsychoPy (v1.78.00, Peirce et al., 2019). A single run consisted of 9 blocks of 40 s each; during 5 blocks neutral pictures were presented, during 4 blocks nicotine-related pictures were presented. Each picture was shown for 4 s. Patients were instructed to look at the pictures. Neuronal response contrasts during tobacco-related cues and neutral pictures were then identified and compared using the multiplanar activation maps calculated in the TBV online analysis: the activation cluster with the most extensive BOLD response to addiction-related information in the ACC, DLPFC, and insula was defined as region of interest for each person and day individually (threshold *t* = 3). The ACC, the DLPFC and the insula were identified on the first acquired EPI image of the online analysis using conventional neuroanatomical MRI landmarks (Ulmer, 2010) and the multiplanar reconstructions offered by TBV, and later validated in the offline analysis after transfer to Talairach space.

(2) *Resting state:* Resting state-sequences were acquired on each day before and after the NF-task: the results of these sequences will be presented in elsewhere (Keeser et al., unpublished).

(3) *rtfMRI NF-paradigm:* The NF-training consisted of three sessions of NF training with three NF runs each. Apart from the NF-task during addiction-related cues, the paradigm of a single NF run was identical to the paradigm of a cue exposure run. During the presentation of tobacco-associated stimuli, participants were instructed to decrease their individual neuronal responses in the target ROI. ROI-based BOLD responses were calculated and visualized using the Turbo-BrainVoyager^3^. The BOLD responses in the target ROI were visualized using a ‘graphical thermometer,’ which based on the top one-third of voxels with the highest *t*-values for BOLD responses for the comparison of addiction-related and neutral stimuli. During the neutral condition, participants were requested to look at the pictures without any further instruction.

Between NF runs, participants of both groups were asked about their perceived success during the rtfMRI training run and received feedback from a staff member. All participants were encouraged to apply various strategies to identify the best individual method. The participants were not instructed to use a specific strategy for modulation. However, it was recommended that they could try methods that have demonstrated to be successful coping with craving in the past.

### Group Therapy

All patients took part in a certified and manualized “smoke-free program” (Kroeger and Gradl, 2007) over 6 weeks, a program based on cognitive-behavioral and motivational concepts. It includes an induction session, 6 group sessions at 90 min and, 2 individual telephone counseling appointments. The treatment can be divided in 3 phases: creation of motivation, preparation of smoking cessation and stabilization. The program focuses on the positive benefits of a nicotine-free life and uses different methods for behavioral change, e.g., psychoeducation, motivational communication, prevention, understanding, and treatment of relapse, etc. At the induction session, patients get an idea of a smoking-free life and information about smoking. The topics of the six group session is “the ambivalence of smoking,” “errors in logic and alternatives,” “preparations of smoking cessation,” “experiences with the smoking stop,” “identity as a smoking-free person,” and “planning the future”^4^.

### MRI Data Acquisition and fMRI Data Analysis

A 3 Tesla Philips MR System Ingenia scanner with echo planar capability (Release 4.1 Level 3 2013-04-05, Philips Medical Systems Nederland B.V.) and a 32-channel phased array head coil was used for imaging. Subjects had to wear ear plugs and headphones for noise protection. We also used cushions in the coil to minimize head movement. A T1-weighted high-resolution 3D data set was acquired for each subject for anatomical referencing. Functional MR data were acquired using an EPI sequence in the identical position as the anatomical images [Field of View: 230 mm × 230 mm × 132 mm; spatial resolution: 3 mm × 3 mm × 4 mm; slice thickness: 4 mm; gap: 0.15 mm; repetition time: 2000 ms; echo time (TE): 35 ms; 25 axial slices].

The results of the resting state sequence will be presented elsewhere (Keeser et al., unpublished).

#### rtfMRI Pre- and Post Data Processing

We used the TurboBrainVoyager (Version 3.0, Brain Innovation, Maastricht, 2011) for the initial processing and real-time analysis as well as the feedback for the participants. For further analysis, raw-data in a DICOM-format were converted into a NIfTI-format using MRIConvert (Version 2.0.7 build 369, University of Oregon, Lewis Center for Neuroimaging, 2013). All subsequent data-analyses of the fMRI sequence were carried out with the BrainVoyager software package (Brain Innovation, Maastricht, Netherlands). In order to reduce relaxation time effects the first 5 images were excluded from any further analysis. The preprocessing of the fMRI data included high-pass filtering (cut-off: three cycles in a time course) to remove low-frequency signal drifts inherent in echo planar imaging. Additionally, a slice scan time correction (cubic), spatial smoothing (Gaussian filter with FWHM 8.0 mm), and a 3D motion correction (trilinear interpolation) were applied. Functional images were transferred to a standard Talairach brain. Significant BOLD activity was determined by a cross-correlation of the pixel intensity of MR images with an expected hemodynamic response function. Voxelwise *t*-tests were used to identify those brain areas where the signal change differed significantly between tobacco-related responses and neutral stimuli. We used the Bonferroni correction at a threshold of *p* < 0.05 to counteract the problem of multiple testing. For each participant the conditions tobacco-relevant pictures and neutral were calculated as regressors.

### Statistical Analysis

Statistical analysis of the questionnaire ratings of patients of abstinent versus relapse group was calculated with SPSS version 23 with a level of significance *p* < 0.05. Because of the small sample size, we first calculated the non-parametric Mann–Whitney-*U* test for independent samples or the Wilcoxon test for dependent samples. In a second step, the two-tailed *t*-test for independent or dependent samples was calculated. If the results of both tests did not differ, the *t*-test were mentioned instead of the non-parametic test. A general linear model with a repeated measure design was calculated in order to compare variations before and after the NF session.

## Results

### Relapse Rates

Ten patients of the real group remained abstinent during the first 3 months after the therapeutic program, 12 patients relapsed. The abstinence rate was 45.5%. Regarding the sham group, 9 patients remained abstinent, 5 patients relapsed. The relapse rate did not differ significantly between groups (*p* = 0.270; Chi-Quadrat test).

### Comparison of Psychometric Data Between Abstinent and Relapse Group on the Day of the First rtfMRI NF Session

The comparison of patients of the abstinent group compared to the relapse group did not show any significant differences regarding verbal intelligence, CO score and personality on day 1. In addition, there were hardly any significant differences regarding psychopathology. A significant difference between groups was only demonstrated in the Anger-In subscale of STAXI (*p* = 0.001). Additionally, differences between groups regarding pack-years or consumption of cigarettes per day did not differ significantly between abstinent and relapse (see Table 1).

**Table 1:** Comparison of the psychometric data of abstinent group vs. relapse group.

Questionnaire	Abstinent		Relapse		*p*-value
			
	*M*	*SD*	*M*	*SD*	
Pack-years	30.20	12.60	19.96	14.61	0.097
Consumption of cigarettes per day	22.00	5.87	17.67	6.21	0.111
C0 score	1.60	1.075	4.67	6.733	0.171
WST	106.30	7.45	112.08	15.22	0.287
Neo-FFI-Neuroticism	18.50	5.87	17.55	6.74	0.734
Neo-FFI-Extraversion	23.60	6.77	27.73	5.18	0.131
Neo-FFI-Openness to experiences	25.10	7.28	27.45	5.56	0.412
Neo-FFI-Compatibility	26.90	5.43	30.36	4.06	0.112
Neo-FFI-Conscentiousness	31.86	4.09	33.71	4.52	0.803
Fagerström	4.70	2.91	5.25	1.49	0.122
BDI	6.90	6.85	4.92	6.96	0.510
QSU-overall	61.70	24.08	71.75	14.37	0.239
STAI-State	36.39	10.30	39.00	6.99	0.497
STAI-Trait	38.11	10.41	35.00	10.55	0.518
STAXI-State	11.44	2.70	11.25	1.77	0.844
STAXI-Trait-Anger	19.22	3.23	18.17	7.31	0.692
STAXI-Anger-Control	23.89	2.93	21.67	5.98	0.319
STAXI-Anger-Out	13.44	2.96	12.92	4.14	0.749
STAXI-Anger-In	19.44	4.28	13.17	3.01	0.001
BIS-11 attention-to-details	25.44	3.59	23.00	4.44	0.272
BIS-11 motoric-impulsiveness	23.89	4.15	22.17	4.15	0.382
BIS-11 coping	23.11	2.68	22.50	4.30	0.659
AQ	65.80	17.63	69.45	11.32	0.622


**M, mean, SD, standard deviation.**

### Changes of Craving: Influence of Groups (Abstinent; Relapse)

The comparison of QSU-overall score revealed no significant difference between pre–post measurements [measurement before/after rtfMRI NF training session; *F*(1,20) = 0.063; *p* = 0.805]. In addition, the interaction effect was not significant [pre–post treatment ^∗^ group: *F*(1,20) = 0.196; *p* = 0.662]. The between groups (abstinent vs. relapse) difference [*F*(1,20) = 0.893; *p* = 0.356] was not significant.

The comparison of QSU-Factor 1 (strong desire and intention to smoke) did not show any significant differences regarding the pre–post measurements [*F*(1,20) = 0.083; *p* = 0.777], the interaction effect [pre–post treatment ^∗^ group: *F*(1,20) = 0.018; *p* = 0.894] and the between groups difference [*F*(1,20) = 0.723; *p* = 0.405].

The comparison of QSU-Factor 2 (anticipation of relief from negative effect with an urge desire to smoke) revealed non-significant differences between the pre–post measurements [*F*(1,20) = 0.063; *p* = 0.805]. In addition, the interaction effect [pre–post treatment ^∗^ group: *F*(1,20) = 0.133; *p* = 0.720] and the between groups effect [*F*(1,20) = 0.280; *p* = 0.603] were not significant.

### Outcome-Based Comparison of Neuronal Responses During the Cue Exposure Task

**FIGURE 2:**
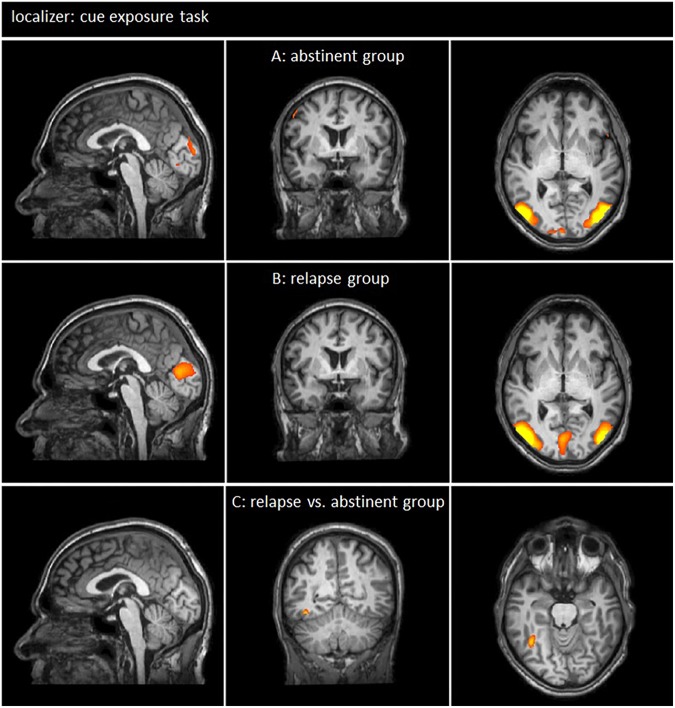
Neuronal responses during cue exposure task [tobacco-related pictures > neutral pictures; *p*(Bonf) < 0.05, *T*-score: 4.830–8]. **(A,B)** Smokers of both groups demonstrated neuronal responses to tobacco related pictures especially in the brain regions associated with visual information processing (*x* = 0; *y* = 0; *z* = 0). **(C)** The comparison of brain responses of the relapse group and the abstinent group showed only very small functional differences within the fusiform gyrus were detected (*x* = –1; *y* = –53; *z* = –18).

During the cue exposure task of the 1st day, smokers that remained abstinent (see Figure 2 and Table 2) and smokers that relapsed within the 3 months intervals after the NF training (see Figure 2 and Table 3) demonstrated tobacco cue-related responses (tobacco-related pictures minus neutral pictures) especially in brain regions that are associated with the processing of visual information (e.g., visual association cortex).

**Table 2:** Neuronal responses in abstinent group during the cue exposure task of the first fMRI session [tobacco-related pictures minus neutral pictures; clusters of >30 voxels, *p*(Bonf) < 0.05, *T*-score: 4.830–8].

Abstinent group								

			Center of gravity			Size	*t*-score	
					
Brain region	Side	BA	*x*	*y*	*z*		∅	Max
**Tobacco-related pictures > neutral pictures**								
Superior Parietal Lobule/Inferior Parietal Lobule	R	7	34	-54	47	6187	6.41	9.67
Superior Parietal Lobule/Inferior Parietal Lobule	L	7	-28	-58	47	2810	5.77	7.77
Middle Frontal Gyrus	R	6	50	5	37	1395	5.36	6.56
Inferior Occipital Gyrus/Lingual Gyrus	L	18/19	-37	-69	-4	15232	8.94	19.25
Inferior Occipital Gyrus/Lingual Gyrus	R	18/19	33	-70	-4	18026	8.77	15.19


*BA, Brodman area; side, hemisphere; L, left; R, right; max, maximal t-score; ∅, average t-score; size, cluster size; voxels, number of activated voxels; x, Talairach coordinate x-axis; y, Talairach coordinate y-axis; z, Talairach coordinate z-axis.*

**Table 3:** Neuronal responses in relapse group during the cue exposure task of the first fMRI session [tobacco-related pictures minus neutral pictures; clusters of >30 voxels, *p*(Bonf) < 0.05, *T*-score: 4.830–8].

Relapse group								

			Center of gravity			Size	*t*-score	
					
Brain region	Side	BA	*x*	*y*	*z*		∅	Max
**Tobacco-related pictures > neutral pictures**								
Superior Parietal Lobule/Precuneus	R	7	29	-54	50	1698	5.59	7.08
Fusiform Gyrus	R	19/37	38	-65	-8	17700	8.90	19.63
Lingual gyrus/Cuneus	R	17/18	2	-80	5	4460	5.50	6.95
Fusiform Gyrus/Middle Occipital Gyrus	L	19/37	-47	-64	-9	11615	8.47	14.52
**Tobacco-related pictures < neutral pictures**								
Fusiform Gyrus/Parahippocampal Gyrus	L	36/37	-24	-42	-14	1582	-6.23	-8.79


*BA, Brodman area; side, hemisphere; L, left; R, right; max, maximal t-score; ∅, average t-score; size, cluster size; voxels, number of activated voxels; x, Talairach coordinate x-axis; y, Talairach coordinate y-axis; z, Talairach coordinate z-axis.*

The comparison of neuronal responses of smokers who relapsed and smokers who remained abstinent revealed only small differences within the fusiform gyrus (see Figure 2 and Table 4).

**Table 4:** Neuronal responses in relapse group minus abstinent group during the cue exposure task of the first fMRI session [tobacco-related pictures minus neutral pictures; clusters of >30 voxels, *p*(Bonf) < 0.05, *T*-score: 4.830–8].

Relapse group versus abstinent group (localizer of the first fMRI session)					

			Center of gravity			Size	*t*-score	
					
Brain region	Side	BA	*x*	*y*	*z*		∅	Max
**Relapse > abstinent (localizer)**								
Fusiform gyrus	R	37	35	-50	-19	498	5.76	7.44


*BA, Brodman area; side, hemisphere; L, left; R, right; max, maximal t-score; ∅, average t-score; size, cluster size; voxels, number of activated voxels; x, Talairach coordinate x-axis; y, Talairach coordinate y-axis; z, Talairach coordinate z-axis.*

### Comparison of the Target ROIs for NF Training Between Groups

The following brain regions were used as target ROIs for the NF training: *abstinent group*: DLPFC left: 2 patients; DLPFC right: 1 patient; insula left: 6 patients; insula right: 1 patient; *relapse group*: DLPFC left: 2 patients; DLPFC right: 2 patients, insula left: 2 patients, insula right: 3 patients; ACC left: 1 patient; ACC right: 2 patients. Overall, we did not find any clear association between treatment success and brain region.

### Functional Variations During Neurofeedback

#### Neuronal Responses of the Abstinent Group During the First NF Session

**FIGURE 3:**
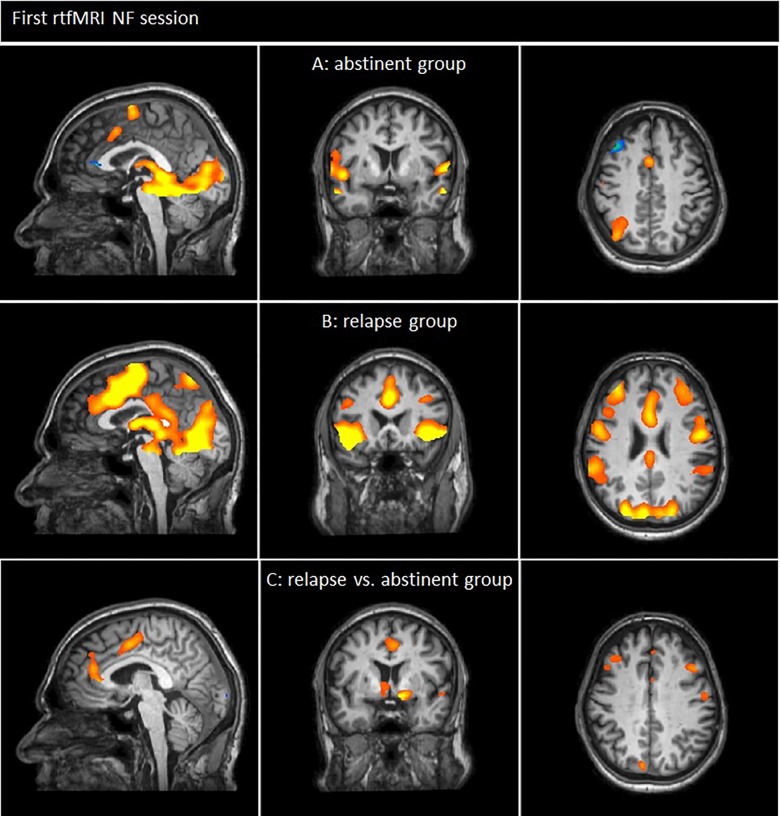
Neuronal responses of the first neurofeedback-session [tobacco-related pictures > neutral pictures; *p*(Bonf) < 0.05, *T*-score: 4.830–8]. **(A)** Smokers of the abstinent group demonstrated enhanced neuronal responses during the presentation of smoking related cues especially in frontal brain regions (e.g., superior/medial frontal gyrus, ACC), the pariatal cortex and the insula (*x* = 0; *y* = 4; *z* = 42). **(B)** Smokers of the relapse group demonstrated enhanced neuronal responses during the presentation of smoking related cues compared to neutral pictures especially in frontal brain regions (e.g., superior/medial/middle frontal gyrus, ACC), the insula, pariatal areas as well as temporal regions and the cuneus/precuneues (*x* = 0; *y* = 18; *z* = 27). **(C)** Neuronal responses of smokers who relapsed compared to smokers who remained abstinent: patients of the relapse group demonstrated enhanced BOLD responses especially in the medial/middle and superior frontal gyrus, the ACC, the caudate nucleus and the superior temporal gyrus compared to patients that remained abstinent. By contrast, the responses in the inferior occipital gyrus and the fusiform gyrus were decreased in the relapse group (*x* = 4; *y* = 3; *z* = 34).

The comparison of BOLD responses during the presentation of smoking-related cues and neutral pictures during the first NF session in smokers who remained abstinent 3 months after the NF training demonstrated increased responses while smoking cues were presented, especially in the superior/medial frontal gyrus, the ACC, the inferior parietal lobule, the culmen, the fusiform gyrus, the superior/inferior parietal lobule, and the insular cortex. Neutral pictures led to increased BOLD responses in the superior/middle frontal gyrus, the ACC and the cerebellum (see Figure 3 and Table 5).

**Table 5:** Neuronal responses in abstinent group during the first rtfMRI NF session [tobacco-related pictures minus neutral pictures; clusters of >30 voxels, *p*(Bonf) < 0.05, *T*-score: 4.830–8].

Abstinent group								

			Center of gravity			Size	*t*-score	
					
Brain region	Side	BA	*x*	*y*	*z*		∅	Max
**Tobacco-related pictures > neutral pictures**								
Superior Frontal Gyrus/Medial Frontal Gyrus	R	6	4	-5	61	3206	6.52	11.34
Medial Frontal Gyrus/Anterior Cingulate Gyrus	R/L	6/32	1	13	40	837	5.33	7.14
Precuneus/Superior Parietal Gyrus/Inferior Parietal Lobule	R	7/40	32	-51	47	4110	5.58	7.00
Superior Parietal Lobule/Inferior Parietal Lobule/Postcentral Gyrus	L	5/7/40	-30	-45	55	2886	2.82	8.65
Fusiform Gyrus/Lingual Gyrus	R	18/19	35	-65	-4	34888	10	25.75
Culmen	R/L		0	-53	-5	51795	7.02	14.29
Fusiform Gyrus	L	37/19	-40	-61	-6	25750	11.02	26.89
Sub-lobar/Insula/Superior Temporal Gyrus/Precentral Gyrus	L	13/22/44	-49	10	3	3564	6.34	10.79
Superior Frontal Gyrus/Middle Frontal Gyrus	R	10	30	49	17	999	5.84	8.36
**Tobacco-related pictures < neutral pictures**								
Middle Frontal Gyrus/Superior Frontal Gyrus	R	6/8	28	18	49	1892	-5.94	-8.14
Middle Frontal Gyrus/Superior Frontal Gyrus	L	6/8	-27	16	51	1217	-5.62	-6.86
Anterior Cingulate Gyrus/Medial Frontal Gyrus	L/R	32/24/9/10	-2	40	9	3982	-6.03	-9.24
Cerebellum	L		-24	-42	-16	1089	-6.74	-10.4


*BA, Brodman area; side, hemisphere; L, left; R, right; max, maximal t-score; ∅, average t-score; size, cluster size; voxels, number of activated voxels; x, Talairach coordinate x-axis; y, Talairach coordinate y-axis; z, Talairach coordinate z-axis.*

#### Neuronal Responses of the Relapse Group During the First NF Session

In the relapse group, NF training during the presentation of tobacco-related cues led to increased responses especially in the ACC, the superior/medial/middle frontal gyrus, the insula, the thalamus, the precentral gyrus, the fusiform gyrus, the inferior parietal lobule/supramarginal gyrus, the inferior/middle temporal gyrus, the cuneus/precuneus, the culmen and the fusiform gyrus compared to the presentation of neutral stimuli (see Figure 3 and Table 6).

**Table 6:** Neuronal responses in relapse group during the first rtfMRI NF session [tobacco-related pictures minus neutral pictures; clusters of >30 voxels, *p*(Bonf) < 0.05, *T*-score: 4.830–8].

Relapse group								

			Center of gravity			Size	*t*-score	
					
Brain region	Side	BA	*x*	*y*	*z*		∅	Max
**Tobacco-related pictures > neutral pictures**								
Cingulate Gyrus/Medial Frontal Gyrus/Superior Frontal Gyrus	L	24/32/6	-1	4	46	29215	7.31	13.7
Precentral Gyrus/Middle Frontal Gyrus	R	6	36	-7	44	11388	5.95	8.11
Middle Frontal Gyrus/Superior Frontal Gyrus	R	6/9	34	33	28	5090	5.83	7.98
Insula/Precentral Gyrus/Inferior Frontal Gyrus	R	13/44/47/22	41	8	1	26082	6.9	14.11
Middle Frontal Gyrus/Superior Frontal Gyrus	L	9/10	-34	35	26	5703	5.64	7.04
Insula/Precentral Gyrus	L	13	-43	5	16	26699	6.86	15.88
Thalamus	L/R		0	-18	2	40474	6.01	10.12
Inferior Parietal Lobule/Supramarginal Gyrus	L	40	-35	-49	39	8650	5.71	8.67
Middle Occipital Gyrus/Fusiform Gyrus	L	19/37	-42	-64	-7	12976	9.16	18.17
Inferior Parietal Lobule/Supramarginal Gyrus	R	40	43	-43	36	12146	6.48	13.29
Cuneus/Precuneus	R	7/31/18	12	-70	30	22047	6.70	12.53
Inferior Temporal Gyrus/Middle Temporal Gyrus	R	37	39	-63	-3	21868	10.78	25.15
Culmen	R		5	-64	-9	20198	8.57	17.44
Cuneus/Precuneus	L	18/31	-14	-74	20	6345	5.88	8.25
Culmen/Declive/Lingual Gyrus	R	18	5	-65	-7	19420	8.45	17.44
Lingual Gyrus/Fusiform Gyrus	R	18	38	-65	-3	23581	10.50	25.15
**Tobacco-related pictures < neutral pictures**								
Fusiform Gyrus/Parahippocampal Gyrus	L	37/20	-26	-44	-14	1530	-6.60	-9.99
Lingual Gyrus/Inferior Occipital Gyrus/Fusiform Gyrus	L	17/18	-14	-89	-6	1878	-6.73	-10.5


*BA, Brodman area; side, hemisphere; L, left; R, right; max, maximal t-score; ∅, average t-score; size, cluster size; voxels, number of activated voxels; x, Talairach coordinate x-axis; y, Talairach coordinate y-axis; z, Talairach coordinate z-axis.*

#### Outcome-Based Comparison of Neuronal Responses During the First NF Session

The comparison of neuronal responses (tobacco-related pictures minus neutral pictures) during the first rtfMRI session of smokers who relapsed and smokers who remained abstinent showed increased responses in the relapse group, especially in frontal brain regions including the medial/middle/superior frontal gyrus, the ACC, the caudate nucleus and the superior temporal gyrus. By contrast, the responses in the inferior occipital gyrus and the fusiform gyrus were decreased in the relapse group (see Figure 3 and Table 7).

**Table 7:** Neuronal responses in relapse group minus abstinent group during the first rtfMRI NF session [tobacco-related pictures minus neutral pictures; clusters of >30 voxels, *p*(Bonf) < 0.05, *T*-score: 4.830–8].

Relapse group versus abstinent group								

			Center of gravity			Size	*t*-score	
					
Brain region	Side	BA	*x*	*y*	*z*		∅	Max
**Relapse > abstinent**								
Cingulate Gyrus/Medial Frontal Gyrus	L	24/6	-2	-2	46	2715	5.63	7.17
Cingulate Gyrus/Medial Frontal Gyrus	R	24/6	7	-70	40	2060	5.96	9.49
Middle Frontal Gyrus	R	8/9	33	27	39	1609	5.88	8.39
	R	8/6	51	8	39	637	5.28	6.13
	R	9	41	21	34	545	5.37	6.28
Anterior Cingulate/Medial Frontal Gyrus	R	32/9/10	8	39	13	3390	5.59	7.36
Middle Frontal Gyrus/Superior Frontal Gyrus	L	10	-31	51	19	940	6.30	9.67
Extra-Nuclear/Lentiform Nucleus/Caudate	R		16	14	0	3852	5.55	7.94
Lentiform Nucleus/Extra-Nuclear/Claustrum	L		-22	12	-2	2261	5.90	10.35
Superior Temporal Gyrus	L	22	-49	13	-4	1117	5.69	8.94
**Relapse < abstinent**								
Lingual Gyrus/Fusiform Gyrus/Inferior Occipital Gyrus/Declive	L	18/19	-29	-74	-8	13679	6.39	11.56
Lingual Gyrus/Inferior Occipital Gyrus/Fusiform Gyrus	R	18/19	24	-85	-10	2263	6.44	8.92


*BA, Brodman area; side, hemisphere; L, left; R, right; max, maximal t-score; ∅, average t-score; size, cluster size; voxels, number of activated voxels; x, Talairach coordinate x-axis; y, Talairach coordinate y-axis; z, Talairach coordinate z-axis.*

## Discussion

The aim of the project was to assess neurobiological response differences between tobacco-dependent patients who benefitted from a combined individualized rtfMRI NF training and group therapeutic program and tobacco-dependent patients who relapsed within the first 3 months after these therapeutic interventions. We focused especially on functional differences between patients of both groups during the first NF training after a general stop of smoking in order to detect early functional features which may be helpful for a fast therapeutic decision-making regarding the application of NF training as add-on-therapy.

For the NF training an individualized target region within the DLPFC, the ACC or the insula was determined for each participant during a localiser run while craving-related tobacco-cues were presented. The selection of the brain region was based on the information from several prior studies showing that these areas are of special importance for cue-elicited craving (McClernon et al., 2005; Wilson et al., 2005; Brody et al., 2007; Goudriaan et al., 2010; Naqvi and Bechara, 2010; Hartwell et al., 2011; Naqvi et al., 2014). The functional localizer for the ROI selection was defined separately for each training session. Reason for this strategy was the consideration that the relevance of functional responses in each brain region can alter during the therapeutic process. This could probably lead to variations in the personal significance of brain regions between sessions. All participants were asked to downregulate craving-related BOLD responses using NF training.

### Clinical Outcome

#### Relapse Rate

The abstinence rate was 45.5% after 3 months. The ‘Rauchfrei Programm’ is the most common cognitive behavioral group program for quitting smoking in Germany (Gradl et al., 2009; Rasch et al., 2010). Former studies indicated that immediately after the program, 60.9% of the participants stopped smoking. After 6 months, the abstinence rate was 40.2%, 31.8% after 1 year (Gradl et al., 2009; Wenig et al., 2013). In our study, the combination of this program with rtfMRI-Neurofeedback did not significantly change the entire abstinence rate. Unfortunately, the relapse rate of the real and sham group did not differ significantly.

#### Craving

The assessment of variations in the clinical data did not show any significant difference regarding craving on the first day. Findings about the association between craving and relapse rates are mixed: a systematic review revealed that (a) there were only a few cases of significant associations between craving collected as part of cue-reactivity studies and treatment outcome, (b) craving after quitting smoking was a stronger predictor of treatment outcome than craving before quitting smoking, and (c) several moderators are likely to influence the relationship between craving and cessation outcome (Wray et al., 2013). The authors conclude that craving is not a necessary condition of relapse. In addition, inconsistent relationships between craving and treatment outcome call the value of craving as a target of treatment into question and emphasize limitations in the prognostic utility of craving (Wray et al., 2013). However, other studies showed that the activation in the right ventral striatum predicted the duration of abstinence beyond the level of nicotine dependence (Owens et al., 2017). Additionally heightened neuronal reactivity in brain regions related to the regulation of emotions, interoception and motor planning/execution to smoking-related cues as well as decreased functional connectivity between insula and cognitive brain areas were presented in subjects that relapsed (Janes et al., 2010). The authors concluded that their data suggest that relapse-vulnerable smokers can be identified before quit attempts, which could enable personalized treatment, improve tobacco-dependence treatment outcomes, and reduce smoking-related morbidity and mortality (Janes et al., 2010).

Despite these inconsistent results regarding the association between craving and relapse, cue-induced craving to smoke has been considered one of the driving forces in continued smoking (Tiffany, 1990). Psychopharmacological interventions have demonstrated only a small impact on cue-induced craving (Tiffany et al., 2000; Ferguson et al., 2009).

### Functional Imaging Data

#### BOLD Responses During the First NF Training Session of the Relapse Group Compared to the Abstinent Group

The comparison of BOLD responses during the NF first training session between patients who relapsed and patients who remained abstinent revealed increased responses in the relapse group, especially in the PFC cortex (SFG/medial PFG/middle PFG) and the ACC (see Figure 3 and Table 7). BOLD responses in the PFC, especially the SFG and MFG, have been related to cognitive processes including executive control (Miller, 2013). The ACC can be attributed to cognitive as well as emotional processes (Miller, 2013): The ACC does have connections to both the ‘emotional’ limbic system and the ‘cognitive’ prefrontal cortex. Thus, the ACC seems to play an important role in the integration of neuronal circuitry to affect regulation (Stevens et al., 2011), including the ability to control and manage uncomfortable emotions. Avoidance of painful emotions is often the motivational force in negative behaviors such as substance abuse. These actions are taken as part of maladaptive approaches to control, avoid, or regulate painful emotions (Stevens et al., 2011). During NF training, a regulation of craving-associated emotions is necessary. Increased responses in the respective brain areas during the NF session in the relapse group could indicate that the downregulation of craving-related responses in brain areas which are especially associated with cognitive and emotional processes is less successful in these patients than in patients who remained abstinent after therapeutic interventions.

Furthermore, increased BOLD responses in the relapse group compared to the abstinent group were shown in the caudate nucleus/claustrum. The caudate nucleus is one of the structures that make up the dorsal striatum which is a component of the basal ganglia (Yager et al., 2015). Apart from various motor functions, the caudate is also one of the brain structures which compose the reward system and functions as part of the cortico-basal ganglia-thalamic loop (Yager et al., 2015). This area has proved to play an important role in the context of dependence disorders, including tobacco. A review of Balfour (2004) indicated that the stimulation of dopamine (DA) projections to the nucleus accumbens play a complementary role in the development of nicotine dependence (Balfour, 2004). The hypothesis in the review proposes that increased extra-synaptic DA in the accumbens confers hedonic properties on behavior, such as smoking which deliver nicotine, and thereby increase the probability that the response is learned. The authors of the review argue that sensitisation of the DA projections to the accumbal core, and the behaviors which depend on this process, play a pivotal role in the maintenance of a tobacco smoking habit (Balfour, 2004). Against this background increased responses in the relapse group during the down-regulation NF task might indicate that these smokers were less able to modulate their neurobiological responses in the reward system which is influenced by dopaminergic innervation. The salience of tobacco-related cues may resist increased in this group compared to smokers who benefitted from the training.

By contrast, the responses in the inferior occipital gyrus and the fusiform gyrus were decreased in the relapse group compared to the abstinent group. These areas have been related to the secondary and tertiary visual cortex and can, e.g., be influenced by attention. Bradley et al. (2003) for example have shown that both extent and strength of functional activity of the occipital cortex were linked to the judged affective arousal of the different picture contents. The author suggested that more extensive visual system activation reflects ‘motivated attention’ where motivational engagement directs attention and facilitates perceptual processing of important stimuli (Bradley et al., 2003). The increased down-regulation of craving-related responses in the relapse group in these areas could indicate that patients of this group chose a different strategy compared to smokers of the abstinent group. Apparently, these patients modulated more strongly visual perception processes which are influenced by motivation, personal significance of the visual information and attention rather than emotional processes or other cognitive processes (including cognitive control, executive functions), related to craving. These modulations seem to be indirect effects since the target ROIs for the modulation were located within the prefrontal cortex/insula.

Craving-related responses between groups before the NF training (during the localizer run) differed only marginally in a small region within the right fusiform gyrus (see Figure 2 and Table 4). For that reason, we assume that differences between the relapse group and the abstinent group during rtfMRI NF cannot be attributed to craving-related responses before training.

#### NF Related Responses During the First NF Session in the Abstinent Group and the Relapse Group

In both groups BOLD responses during the NF trials (presentation of tobacco cues and NF information) compared to the neutral condition during the first NF training sessions were enhanced, especially in brain areas related to cognitive and/or emotional craving processes as well as attention/motivation (see Figure 3 and Tables 5, 6) including frontal/fronto-central areas, the insula, parts of the secondary visual processing system and brain regions which are important for reward processing.

In both groups, real NF had been provided and participants had been instructed to downregulate their brain responses in individually defined ROIs within the frontal cortex. Nevertheless, in both groups brain responses in most parts of the brain were increased during NF compared to the neutral condition. These results may indicate that craving-related responses stayed visible despite NF modulation. Less prominent differences between the cue-related stimulation plus NF and the neutral condition could be expected after several NF sessions (Ruiz et al., 2013b).

Participants who remained abstinent after the training also revealed decreased responses in several frontal areas, including the middle FG, the SFG and the ACC/medial FG. These patients seemed to be able to reduce their individual responses more strongly than patients of the relapse group.

### Comparison of Psychometric Data of the Relapse Group With the Abstinent Group

An array of questionnaires and ratings were used in order to compare psychopathological aspects and aspects of the personality of patients who remained abstinent with those who relapsed. We did not find any differences regarding physical dependence (Fagerström test), pack-years, number of cigarettes per day, intelligence (WST), personality (Neo-FFI), craving (QSU-G), anxiety (STAI), impulsivity (BIS), and aggression (AQ). Concerning anger expression (STAXI), the anger-in subscale demonstrated an increased score on the day of the first rtfMRI NF session for patients who remained abstinent during 3 months after the NF training compared to patients who relapsed. This may indicate a varying approach in both groups regarding the expression of negative emotions. However, the difference seems to be small: The results of all other subscales were comparable between groups. Altogether, the differences between groups were marginal and we did not detect any reliable variables influencing the success of the therapeutic approach.

### Individualized ROI Definition

The selection of a personalized ROI enhances the probability for valid feedback: the selection of the target ROI was based on each individual’s neuronal responses during the localizer run during the presentation of craving-related information. The individualized ROIs which were identified for feedback encompassed areas of the DLPFC, the insula or the ACC. These regions were selected based on information from former studies using exposure to smoking-related cues compared with neutral cues (Janes et al., 2016, 2017). In these studies the ACC has been reported to be involved in executive functioning such as decision making, choosing between alternatives and evaluating possible outcomes to optimize results. In addition, the ACC is an important area for emotional processing (Bush et al., 2000). Furthermore, previous rtfMRI NF studies have demonstrated that the activity within the ACC can be influenced by the participants (Weiskopf et al., 2003; Hamilton et al., 2011; Hartwell et al., 2016).

The challenge of not smoking following exposure to smoking-related cues presents both a cognitive and an emotional task for nicotine-dependent smokers while individual variations in the involvement of the ACC and the PFC is expected (Hartwell et al., 2016).

The selection of an individualized task-driven NF minimized the risk of providing NF from a non-activated area, e.g., owing to possible alterations as a result of previous NF sessions (Hartwell et al., 2016). It seems sensible for future studies to include the examination of an optimal target region for NF in patients with tobacco dependence.

Disadvantages of an individualized task-based ROI definition may be that the anatomical specificity is reduced and the possibility to compare the results between patients is limited.

### Comparison With the Results of Other NF Studies With Patients With Tobacco Use Disorder

In some aspects the design of the present study was comparable to the design of the study of Hartwell et al. (2016). In both studies an individualized NF approach for craving-associated BOLD responses was used, based on an initial run during which smoking-related cues were employed to provoke craving; participants completed three neuroimaging visits with three NF runs each. The results of the study of Hartwell et al. (2016) reveal decreased subjective craving and cue-induced brain activation.

The results of the present study show small differences in terms of craving between groups: the difference reached trend level. In addition, BOLD responses were influenced by the day of measurement and the group. One main difference between these studies is the inclusion criteria: unlike our study Hartwell et al. (2016) did not include treatment seeking smokers.

### Influence of Intensity of Craving on BOLD Responses

The study of Wilson and Sayette (2015) demonstrated that brain responses measured during mild states of desire fundamentally differ from those measured during states of overpowering desire (i.e., craving) to use drugs (Wilson and Sayette, 2015). A meta-analysis revealed that fMRI cue exposure studies using nicotine-deprived smokers produced different patterns of brain activation to those using non-deprived smokers (Wilson and Sayette, 2015). The authors conclude that the intensity of the urges does matter, and more explicit attention to urge intensity in future research should have the potential to yield valuable information about the nature of craving (Wilson and Sayette, 2015). In our study, the intensity of craving was comparable between groups: we did not find any differences concerning the craving intensity between the abstinent and relapse group.

### Limitations

Several limitations should be noted in the interpretation and application of the results.

Our interpretation is based on the results of the real group and does not consider the results of the sham group that will be reported elsewhere in detail (Karch, Paolini et al., unpublished). Therefore, even if our results are suggesting neurofeedback specific effects, we cannot completely exclude that some of our findings are independent of the targeted NF approach. Various control groups, for example with alternative – not neurofeedback based – strategies are needed, perhaps in future studies with bigger sample sizes to address this question.

Concerning the paradigm, we did not include any cue-exposure scanning without feedback for training evaluation directly after the rtfMRI training. In addition, we did not record neurobiological data during the post-training survey 3 months after the NF sessions. For that purpose it is not possible to determine whether and to which extent neurobiological effects are enduring. During the rtfMRI training, tobacco-associated pictures as well as neutral pictures were presented repeatedly to the participants. This might have led to some kind of habituation and a diminished response at later repetitions. However, we expected that habituation effects would occur in the relapse group as well as in the abstinent group.

Future research in treatment-seeking smokers prepared to initiate a cessation attempt is needed and should include further fMRI sequences after the NF training in order to assess the persistence of neuronal effects.

The optimal number of NF training sessions is not yet clear. In the present study, all patients participated in three rtfMRI NF sessions in order to enhance the power compared to single session training. However, further studies are needed focusing on the systematic examination of this topic.

## Conclusion

Patients with tobacco use disorder who remained abstinent for at least 3 months after a behavioral group therapy combined with a rtfMRI NF training demonstrated decreased neural responses during the first cue-associated NF training session compared to patients who relapsed, especially in the ACC, the SMA as well as dorsolateral prefrontal areas. It seems that a pronounced neural reduction in frontal brain regions related to cognitive-emotional processes during craving in the first NF training may be used as an early predictor of a better therapeutic success for quitting smoking in patients with tobacco use disorder. As our NF target areas, i.e., the ACC, the insula and the DLPFC, were mainly included in these brain areas of decreased neural responses, the success in smoking cessation may be related to the success in conducting effective rtfMRI NF. This approach needs further research, exploration and development, especially in order to assess the persistence of neuronal and therapeutic effects.

## Data Availability

The datasets generated for this study are available on request to the corresponding author.

## Author Contributions

SK, MP, ArR, BE-W, OP, DK, and TR conceived and designed the experiments. The experiments were performed by MP, SG, HJ, AnR, OY, BR, and DK. Data analysing was done by SK, MP, SG, HJ, AnR, MM, CF, BR, AC, ArR, and DK. SK, MP, MM, CF, BE-W, OP, DK, and TR contributed in the writing of the manuscript.

## Conflict of Interest Statement

The authors declare that the research was conducted in the absence of any commercial or financial relationships that could be construed as a potential conflict of interest.

## Footnotes

1 http://www.who.int/mediacentre/factsheets/fs339/en/

2 http://csea.phhp.ufl.edu

3 http://www.brainvoyager.com/TurboBrainVoyager.html

4 https://www.rauchfrei-programm.de/images/Rauchfrei_Jahresbericht_2018.pdf
